# “Difficult” dental patients: a grounded theory study of dental staff’s experiences

**DOI:** 10.1038/s41405-022-00115-7

**Published:** 2022-08-08

**Authors:** Adam Alvenfors, Mersiha Velic, Bertil Marklund, Sven Kylén, Peter Lingström, Jenny Bernson

**Affiliations:** 1 Department of Cariology, Institute of Odontology, Sahlgrenska Academy, University of Gothenburg, Gothenburg, Sweden; 2 Public Dental Service in Region Västra Götaland, Folktandvården Trädgårdsgatan, Skövde, Sweden; 3 Public Dental Service in Region Västra Götaland, Folktandvården Sylte, Trollhättan, Sweden; 4 Department of Primary Health Care, Sahlgrenska Academy, University of Gothenburg, Gothenburg, Sweden; 5 Research, Education, Development & Innovation, Primary Health Care in Region Västra Götaland, Vänersborg, Sweden; 6 Department of Behavioral and Community Dentistry, Institute of Odontology, Sahlgrenska Academy, University of Gothenburg, Gothenburg, Sweden

**Keywords:** Dental psychology, Dental public health

## Abstract

**Introduction:**

The “difficult” patient is a well-known and potentially negative character in various care contexts.

**Objectives:**

This study aimed to generate a conceptual framework explaining the main concerns about “difficult” dental patients, and obtain a deeper understanding of their characteristics, how they affect the dental staff and how the staff think and act in order to manage these patients.

**Methods:**

Ten interviews were conducted with professional dental caregivers, including dentists, dental hygienists, and dental nurses. The audio-recorded interviews were transcribed and analyzed in accordance with the principles of grounded theory.

**Results:**

The main concern regarding “difficult” dental patients generated a framework of seven descriptive interrelated lower-level categories grounded in the data, subsumed in the core category “*balancing subjective difficulties”*. The informants perceived the possession of “*showing interaction difficulties*” and “*having bio-psycho-social complexity*”, as characteristic features of “difficult” patients, who could further adversely affect the dental staff by “*evoking negative emotions and behaviors*”, “*hampering self-esteem and job satisfaction*”, and “*impairing life and health in general*”. To manage the dental care of these patients, the staff used strategies aimed at “*activating internal and external resources*” and “*creating adaptive treatment relations*” with patients.

**Conclusions:**

The dental staff’s meaning of the phenomenon of “difficult” dental patients points to specific characteristics, effects, and handling strategies. The core category captures the contradictory dynamics of characteristics and affects as these concepts seem interrelated to the caregivers’ handling capacity. The dental staff’s possibility of handling the main concern through *balancing subjective difficulties* depends on contextual conditions regarding time, to bring the patient and/or oneself at the center of attention. This indicates a need for further research regarding dental interactions and studies generalizing the outlook on “difficult” dental patients.

## Introduction

The “difficult” patient is a well-known character that appears in various health care settings, such as health care in medicine [1, 2], mental health care [3–7], and dental health care [8, 9]. In the arena of health care in medicine, at least 15–30% of patients are perceived as “difficult” in some respects [1, 10–13]. While the prevalence of “difficult” patients in dental health care is more uncertain, there is one study that indicates an estimated challenging patient rate of 25% [14].

There are no concrete definitions of “difficult” patients, and the labeling of such patients has varied from hateful [15], difficult [1], to challenging [14]. Previous research tends to focus on the inherent problems of “difficult” patients. “Difficult” patients in mental health care have been associated with specific diagnoses, such as paranoid psychosis, chronic depression, substance abuse, and personality disorders, but also with difficult behaviors, as a result of which they are perceived as demanding and claiming, attention-seeking and manipulating, aggressive and dangerous, and withdrawn and hard to reach [4]. Further, “difficult” patients in the field of health care in medicine have been characterized by psychosomatic symptoms, personality disorders, and somatization [11, 12].

The characteristics of “difficult” patients in dental care are still unknown, although aggressive behaviors and dental anxiety among patients may have a negative impact on dentists regarding burnout and stress [14]. Further, for dentists, patients’ aggressive behavior has been found to be the most challenging trait to handle [14].

Previous research may be viewed as somewhat single-sided; this also seems to place the responsibility of the experienced “difficulty” on naturalistic factors originating from the patient himself. However, some recent explorations of “difficult” patients in dentistry emphasize the impact of complexity as a predictor of encounters perceived as difficult [13, 16]. A study conducted in mental health care showed that perceived “difficulties” differed between different groups of patients, professional caregivers, and experts. “Difficult” patients in mental health care saw themselves as individuals in need of help and predominantly blamed professionals for not being sufficiently understanding [6]. Professional mental health caregivers, on the other hand, felt that patients’ large amounts of complex and often coherent problems were the cause of difficulties [6], while mental health care experts highlighted professionals’ pessimism and patients’ help-seeking styles as important explanatory factors for the difficulties experienced [6].

Health care providers in medicine have been shown to struggle with care for “difficult” patients as they induce emotions of frustration [17], or even hatred toward patients [15]. “Difficult” patients have also been associated with more diagnostic errors by doctors [18] and less patient satisfaction [19]. Similar effects of stress and frustration have been reported by caregivers in mental health care [3–7, 20].

In dentistry, “difficult” patients constitute a stress factor for dentists [8, 9, 14], although their overall effects on the dental staff and dental interaction are unclear. However, in a study of interactions between dentists and patients, it was found that the dentists’ intuition and judgment influenced the desired health outcome and the possibility to modify the treatment plan [21]. Other studies indicate that the type of dental care provided could be influenced by the relationship between the therapist and the patient [22, 23] and that the dentist’s perception of the patient could influence the quality of restorative services provided [24].

Taking recent studies into account [6, 13, 14, 16], one may argue that the “difficult” dental patient is a triadic rendezvous between caregiver, patient, and the clinical context of care. Understanding what factors are perceived as “difficult”, can be of importance to assist our profession to make full use of the care available. In addition, the Declaration of Human Rights establishes the right to good health and equal care performed with respect for the patient’s self-determination and autonomy, characterized by good communication and sensitivity to the patient [25]. Future qualitative research exploring the phenomenon of “difficult” patients is needed to shed light on previous aspects where quantitative methods have fallen short. For example, investigate the dental staff’s resilience to these patients.

This grounded theory study aimed to generate a conceptual framework explaining the main concern about “difficult” dental patients and thereby gain a deeper understanding of their characteristics, how they affect dental staff and how dental staff think and act in order to manage dental care for these patients.

## Materials and methods

Grounded theory [26] was chosen as the research framework since it comprises a well-suited design for exploring human social processes, discovering new theories from data, and determining possible solutions to a main problem [27]. This approach involves methods related to the recruitment of informants, data collection, and analytical procedures, where the characteristic simultaneous collection and analysis process influences further data collection and possibilities to explore specific theoretical links in order to achieve the quality and saturation of the data.

### Participants

This study’s participants included eight women and two men aged between 24 and 67 years, comprising five dentists (including the male participants), two dental hygienists, and three dental nurses. The selection of informants was conducted with aim of obtaining a large variation in the data. Informants with different work experiences, sub-specializations, and career paths were strategically selected from nine different clinics. The inclusion criteria were permanent employment and being professionally active in the public dental service in the Västra Götaland region of Sweden. Upon request for participation in the study, the participants received both oral and written information about the study’s purpose and its confidentiality. All the interviewed respondents were willing to participate in the study and gave written consent.

### Data collection

The interviews took place in a conference room at the Department of Cariology, University of Gothenburg, or at the informants’ workplaces or homes, and lasted 25–60 min. The interviews adopted a conversational style [28, 29], with the following three questions formulated in advance:

1. Which patients do you find to be difficult, and what is characteristic of them?
2. How do they affect you as a dental health care provider?
3. How do you think or act to handle and treat these patients?

The above questions were followed by relevant probing and follow-up questions. Each interview was audio-recorded and transcribed verbatim by the interviewer for sequential analysis together with the final author (JB). The emerging preliminary results of the analysis directed the data that were needed next, and theoretical sampling that aimed to saturate the emerging categories through additional information was conducted. After eight interviews, saturation of the data was observed: Two supplementary interviews were conducted, and when these interviews did not generate any additional data, it was decided that saturation had been achieved.

### Data analysis

Initially, an open line-by-line coding was performed, where substantive codes reflecting the meaning of the data were identified and labeled concretely. Emerging codes with similar meanings were clustered into more comprehensive categories and labeled on a more abstract level. Each category was saturated with the underlying properties or subcategories identified in the data. A core category that was central to the data and that explained the main concern was identified. The last step in the analysis was to explore relationships between the core category and the subcategories, using theoretical abstraction to explain how the main concern was managed, and in which the researchers moved between inductive and deductive thinking. Memos written during the entire process and containing preliminary assumptions, ideas, and theoretical reflections were also examined.

The simultaneous collection and analysis of data were performed between March 2018 and April 2019 by two of the authors (AA and MV), with supervision by the final author (JB). All authors have previous but different experiences from dentistry: AA as a dental student at the end of their training, MV as a dental hygienist with approximately five years’ work experience, and JB as a dentist with over ten years’ experience of general dental care and approximately 15 years’ experience in oral medicine and the treatment of dental anxiety and phobia. Regarding the three other co-authors who were involved in compiling the analysis, the first co-author (PL) is a specialist in cariology with many years of experience in caries treatment, the second co-author (SK) is a psychologist who has a limited knowledge of dental care, and the third co-author (BM) is a specialist in general medicine with no professional experience of dental care. All authors strived to avoid being consciously governed by their own pre-structured understanding and to maintain a self-reflective attitude to ways in which the research process could be influenced and how this, in turn, could influence the researcher.

### Ethical approval

The Swedish Ethical Review Authority granted ethical approval for this study, application number 635-18.

## Results

**Fig. 1:**
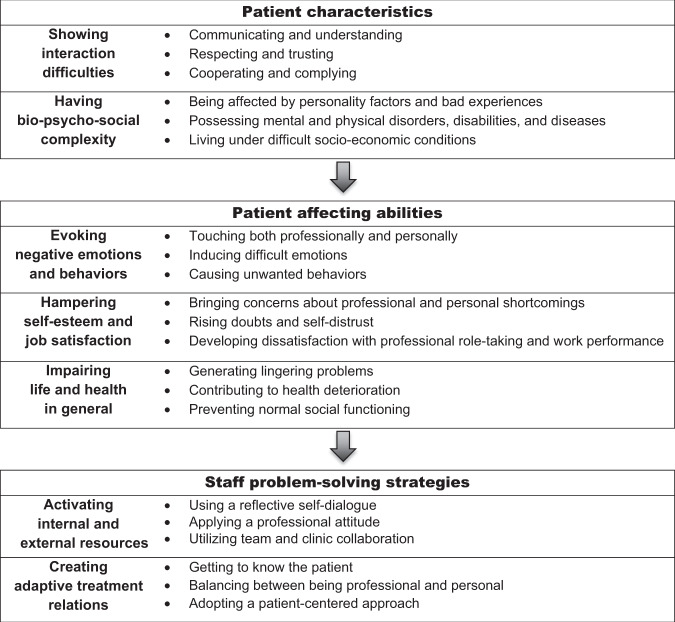
Dental staff’s experiences of “difficult” dental patients. A grounded theory hierarchy of patient characteristics, patient affecting abilities, and the staff’s problem-solving strategies.

A conceptual framework was generated where the core category “*balancing subjective difficulties*” emerged. The core category has the strongest explanatory power at the most abstract level of the main concern. This core category was generated from seven interrelated lower-level categories grounded in the data describing the characteristics of “difficult” patients, including “*showing interaction difficulties*” and “*having bio-psycho-social complexity*”; their affecting abilities, including “*evoking negative emotions and behaviors*”, “*hampering self-esteem and job satisfaction*”, and “*impairing life and health in general*”; and the staff’s problem-solving strategies, including “*activating internal and external resources*” and “*creating adaptive treatment relations*”. This framework formed a pattern with the underlying descriptive subcategories (Fig. 1) illustrated by quotes from the participants in the following text.

### The core category: balancing subjective difficulties

The core category *balancing subjective difficulties* encapsulates the one-sided perception of the two-sided interaction between the dental staff and “difficult” dental patients.

**Fig. 2:**
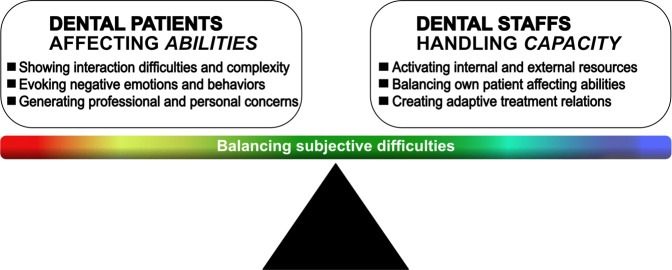
Balancing subjective difficulties. The core category and suggestions for problem-solving processes.

In this study, balancing subjective difficulties means that the “difficult” patient characteristics and affecting abilities are dynamic concepts interrelated with the individual caregivers’ abilities and capacity to deal with this impact, illustrated in Fig. 2 by a balance board. Further, participants adjusted to such subjective “difficulties” by inducing problem-solving strategies aimed at overcoming the perceived difficulty. Thus, the dental staff’s possibility of balancing the perceived difficulties depended on contextual conditions regarding sufficient time, to bring the patient and/or oneself at the center of attention. Balancing the difficulties captures the art of resilience occurring both before, during, and after a “difficult” patient encounter. The main point taken by the participants is the ability to balance the consequences and characteristics of a challenging encounter and, in turn, how the experienced difficulties may even be interrelated to caregivers’ own handling capacity.

### Patient characteristics

#### Showing interaction difficulties

- Communicating and understanding
- Respecting and trusting
- Cooperating and complying

“Difficult” patients were perceived as being unable to communicate and understand, which generated difficulties in interacting with them. This may be caused by misunderstandings caused by language difficulties, cultural differences, or other barriers to communication: “*Some cultures have a different world of concepts and different ideas about the causal relationship with diseases*”. Further, the staff described difficulties in communicating with patients who, for various reasons, used an interpreter or close relative as a communicator: “*The patient comes with her husband, and the husband speaks for his wife. I want to talk to the person sitting in the chair, but then someone else answers. It is their culture, and I cannot change that, but I must try to relate to it. Try to understand it*”. Communication problems might also be due to mental or cognitive dysfunctions or mental illness, for example, depression or Alzheimer’s disease: “*I feel that the patient cannot accept what I am saying… I am informing [them], but they do not understand*”. It may also be difficult for patients who are afraid and anxious to express their feelings and needs: “*[There are] those who just lie there, and you can’t reach them. They are the most difficult to treat*”.

The dental staff found that a lack of respect and trust for their professional know-how and poor acceptance caused difficulties when providing information, reaching a therapy decision, or performing treatment. One interviewee stated, “*When I feel that the patient has made up their mind and does not really listen … I still think I have the knowledge and maybe the right answers … But I am not listened to because they have a different opinion that [they think] is superior to mine. It is difficult to find common ground*”.

The participants highlighted the importance of showing respect for patients’ self-determination and integrity; however, they noted that patients’ choice of care could not involve therapies that were not individually or odontologically indicated: “*The difficulty lies in the fact that they [the patients] want to steer it in a direction that you don’t think is right*”. The staff also emphasized that it could be difficult to treat another professional care provider when it came to authority and creating trust and confidence in their professional dental knowledge: “*[They] feel that diseases do not affect them, [that] they can do as they please because doctors do not get caries*”.

A lack of patient cooperation during treatment constituted a source of frustration for the caregivers, who put a lot of effort into meeting the patients’ needs and requirements when they tried to facilitate their treatment: “*We help and support them along the way, but they must understand that their role is decisive for the outcome and what the treatment result will be*”. Similarly, *non-compliance* with advice and instructions was perceived as frustrating and energy-draining when great efforts were made to ensure the patients’ understanding of information and the need for a change of habits*:* “*Patients with mental illness may have [neglected their] oral hygiene for six months, and so what we have built up is destroyed. So, you have to change their therapy again*”. In addition, the lack of patient-centered collaboration with other care providers or close relatives was difficult for the caregivers to work with and overcome. An interviewee recounted, “*When parents come with their children, and [the parents] just sit there with their mobile phones. ‘Be supportive of your child and more helpful during treatment!’*”

#### Having bio-psycho-social complexity

- Being affected by personality factors and bad experiences
- Possessing mental and physical disorders, disabilities, and diseases
- Living under difficult socio-economic conditions

“Difficult” patients were perceived to have a “difficulty” within themselves or in the surrounding world, in both the present and/or past, which required time and focus to embrace and understand. The whole or parts of this complexity formed a background of underlying explanations of the patients’ current problems and symptoms. An interviewee explained, “*You have to go through all the medications to make sure they are right. Then they might want to talk. Sometimes they show a lot of emotions. They might have been through something really bad… it takes the energy out of you… it’s difficult when they open up too much… and then you have to start working… you take up the saliva ejector… start reclining the dental chair… ‘and now we have to work a little!*’”

Personality factors such as high vulnerability could affect patients’ experiences and ability to handle stressful situations, resulting in challenging communication styles. An interviewee recounted, “*He threw his wallet on the counter, he had money and wanted to fix all his teeth. He told us that he had been in jail… he felt threatening… but we solved it…we referred him to a prosthetist*”.

The participants also described difficulties when treating patients who had mental and physical disorders, disabilities, and diseases, for example, social dysfunction, injuries, infections, autoimmune diseases, and degenerative diseases. This, in turn, necessitated that the dental staff collaborate with other care providers linked to the patient and their close relatives: “*They may be in need of a multimodal treatment approach. I remind them that ‘I can take care of this [the mouth], and I can listen to what you have to say. But for your other needs, I will refer you to other professionals.*’”

The complexity further included living under difficult socio-economic conditions, for example, living with loneliness or social isolation, poverty, unemployment, problems connected to immigration, substance abuse, and poor living habits. Complexity arises when many underlying factors interact, which requires time and focus to understand. As one interviewee noted, “*Those who talk a lot can be a difficult patient group. Those who have a lot inside and want to share their story. It can be hard for me to just receive all of this*”.

### Patient affecting abilities

#### Evoking negative emotions and behaviors

- Touching both professionally and personally
- Inducing difficult emotions
- Causing unwanted behaviors

The participants understood that they were affected both professionally and personally by unfair and disrespectful interactions. The patients affected the caregivers by inducing difficult emotions such as frustration, discomfort, anger, anxiety, hopelessness, powerlessness, and despair: “*When I am treated very disrespectfully, it arouses aggression in me. It is a challenge to handle*”. These emotions caused unwanted behaviors within the caregivers, such as irritation, reluctance, inflexibility, and disengagement, which could consequently have a negative impact on the patients’ treatment and affect the staff professionally: “*It then becomes an interplay that does not create suitable conditions for good treatment*”. The perceived negative effects varied in intensity and duration depending on the caregivers’ personal resources and earlier experiences of treating “difficult” patients: “*We experience difficult patients in different ways*”. Thus, these difficult emotions and consequences could be more or less difficult to process and handle, and the unwanted behaviors could be more or less difficult for the staff to prevent and hide from the patients. One interviewee explained potential contextual shortness of time touching staff both professionally and personally: “*For the first five minutes, you might think it is… great… But then you get frustrated and stressed because you feel that there is no time for what you intend to do*”.

#### Hampering self-esteem and job satisfaction

- Bringing concerns about professional and personal shortcomings
- Raising doubts and self-distrust
- Developing dissatisfaction with professional role-taking and work performance

Showing anger and frustration toward the patients conflicted with the dental staff’s perceptions of what constitutes a good professional caregiver. This responsive behavior induced concerns about their professional and personal shortcomings when handling “difficult” patients: “*How did the patient behave? What could I have done better? What went well?*” This raised doubts and self-distrust about their own abilities and a fear of failure, which hampered the caregivers’ self-esteem and job satisfaction. One interviewee explained, “*It is always traumatic not to succeed. You can handle a lot of difficulties and time-consuming treatments if the procedure ends well. It is like you have it in you that you should succeed*”. Although the staff strived to be professional in their performance, they described difficulties in balancing being both professional and personal in their interactions with patients. This leads to dissatisfaction with professional role-taking if they feel rushed or badly treated: “*It is difficult to always be as professional as you have imagined. Of course, you get affected. It would be a lie [to say] otherwise. We are emotional people, and we have emotions*”.

#### Impairing life and health in general

- Generating lingering problems
- Contributing to health deterioration
- Preventing normal social functioning

The staff’s encounters with “difficult” patients, the consequent negative impact on their emotions and behaviors, and the additional hampering effects on their self-esteem and job satisfaction led to long-term stress problems that not only affected the staff’s working ability but also their lives and health in general: “*These patients can be fun to work with, but they can crack you, too, if there are too many*”. “Difficult” patients had an influence on the staff not only during the dental work but also after their shifts were completed. The caregivers described difficulties related to not bringing home patient-related stress and a continued lingering of problems that resulted in unwanted thoughts that were difficult to escape from: “*I can bring things home and think of situations that have happened. You try to disconnect, but it happens anyway*”. These stress-related lingering concerns were perceived by the participants to create anxiety and disengagement, followed by tiredness and privacy-seeking. Accumulated stress among the caregivers from encounters with “difficult” patients could entail withdrawal from enjoyable activities: “*I can’t be nice to others. I can’t be around people. Sometimes I can’t call my friends when I get home, I don’t want to talk to anybody at all*”.

Moreover, “difficult” patients were perceived to have the potential to affect the staff during spare time and prevent normal social functioning: “*Patients sometimes approach me [in the neighborhood] and point at something [in their mouth] or articulate frustration [about their teeth]*”.

### Staff’s problem-solving strategies

#### Activating internal and external resources

- Using a reflective self-dialogue
- Applying a professional attitude
- Utilizing team and clinic collaboration

To manage and regulate their reactions and emotions in the reception and understanding of “difficult” patients’ expressions, the participants described an internal process in which they used a reflective self-dialogue, which included self-control, emotion-regulation, and perspective taking. This process helped the caregivers maintain focus and empathy for the patients without personally absorbing their communications: “*You have to talk to yourself. Even though you know the patient is anxious, it is still difficult to [deal with] very rude people. It requires much internal monologue*”. In order to deal with the lingering effects of inner dissatisfaction, anxiety, and discomfort, the participants described an internal process in which they reviewed the difficulties. This process included reflection and learning, after which opportunities arose to release themselves from the problem and move on: “*Think through it, learn from it, and then release it*”. Finally, to achieve a balance between their work and private lives, the caregivers strived for a normal life where they could leave patient-related problems at work. They tried to rest, exercise, and sleep well: “*What I like best is just to be or to walk in the forest, where it is very quiet*”.

Dealing with difficult patients required stability, self-awareness, and becoming comfortable with failures. This was perceived to develop through accumulated experience: “*I remember how I first experienced my profession and my professional role …, back then I thought I knew everything, I was everyone’s savior. However,… then you learn a process where you learn from your mistakes, it doesn’t always go that well*”.

Awareness of potentially risky interactions could prevent “difficulties” from appearing if the dental staff were allowed to reflect, properly prepare for the care in advance, try new approaches, and work in a structured manner. As one interviewee mentioned, “*At the beginning of my career, I looked in the booking calendar at what would be done technically on the next day. Now, instead, I look in the booking calendar at* who *is coming*”. This placed demands on the staff to apply a professional attitude, not only in front of the patients but also in terms of their own internal management of the patients and of the patients’ impact on their self-esteem, emotions, and behavior: “*You do not go on the counterattack with the same emphasis, or without dampening it down a bit. [You say] ‘These are my professional boundaries.’*” The staff’s professional attitude toward themselves was important in order to avoid professional failures becoming negative personal experiences.

Treating “difficult” patients also required an intensive focus on the patient during conversation and treatment, which required working conditions that facilitated this focus in terms of time and not being disturbed: “*You must be given the time you need to resolve such situations*”. To be able to recharge and regain the energy required for professional attention and interaction with the next patient, the caregivers also needed time to prepare and reload “*so [that they did] not carry the feelings from the previous patient to the next one*”. The need to receive enough time for care performance and preparation was, thus, conveyed; this time cannot be charged for directly but may be profitable in the long run. In connection to receiving enough time, the staff expressed hopes for a higher value of care quality and fewer demands on hourly rates: “*Care cannot be evaluated only in economic terms*”.

When the participants sensed that their professional roles were being taken advantage of, they worked in a more intertwined and structured manner. When the team worked well, the participants experienced good opportunities to use the team’s competence and different working role opportunities: “*We are a team in the room …, so we should try to support each other in what others do not consider*”. According to the participants, good team interaction provided opportunities to jointly coordinate and process experiences of patient behaviors, which created common perspectives and increased understanding. This further encouraged trust and confidence in the patients: “*The patient senses the relationship between the dentist and the dental assistant. Everything will be much easier if the team is working well together. Because then the patient also feels safe*”. The importance of accessing support from the team was highlighted: “*Having someone direct, being allowed to talk about how it was. You want to share it with someone, and you have to do that to get better for the next time*”. In addition, the dental staff reflected upon the importance of support and guidance from an outsider so that having difficulties with a patient would not be perceived as a weakness or a failure at the clinic: “*I think maybe it would be good for many clinics if someone came, so we could discuss issues that are difficult and patient-related*”.

#### Creating adaptive treatment relations

- Getting to know the patient
- Balancing between being professional and personal
- Adopting a patient-centered approach

The dental staff emphasized the importance of the first meeting with a patient, which contributed to communication adaptation and the framing of what followed. They discussed the importance of showing interest in the patient and spending time listening to them from the beginning. By getting to know the patient, they found that they took part in a beneficial familiarization process in which they gradually learned more about the patient. An interviewee recounted, “*I welcome them [in the waiting room] and introduce myself. Sometimes it’s a bit of a walk [to the treatment room]… then you start chatting… get [the patient] to start talking by telling a bit about yourself*”.

This process involved first exploring safe and neutral conversation points to make the patient more relaxed and open to future important communication, such as anamnesis, information, and therapy discussions and planning: “*We have often joked a bit in the treatment room and talked quite a lot with the patients, not just about [their] care*”. To take a detailed dental history and build an adaptive treatment relationship, conveying empathic understanding and security was critical: “*It is still important to create a feeling of security and understanding for the patient, [letting them know], ‘I see you, I hear you.’*” The staff perceived that such interactions also included non-verbal communication, which played an important role in the communication of understanding that formed the basis for building mutual trust and respect during treatment.

In this communication, the staff described the benefit of balancing between being professional and personal since this made it easier to connect with and understand the patients. When balancing these interactions, the staff adopted their own personal skills and revealed select parts of their private selves, without at the same time significantly compromising their professional attitudes toward the patient*:* “*If you know the patient or something about their interest, talk a few words about that… try to approach them a little bit*”. This balancing gave the interaction a lighter and a more pleasant undertone and was perceived as facilitating the understanding of the patients’ treatment goals and expectations for dental care. This balancing act was experienced to prevent potential future difficulties, but also to reduce the effect of previous negative challenges. The interaction could then be deepened through increased trust, respect, and reciprocity. At the same time, it was important to balance their personal expressions with a professional approach in order to create an adaptive and respectful treatment relationship: “*I think a big part of achieving successful treatment is building a relationship with the patient. Because they may have to come regularly, quite intensively over a period of time*”.

By adopting a patient-centered approach by including patients in therapy discussions and decisions, patient collaboration was created, which made the treatment process and relating risks clear from the beginning: “*Information first, because afterward, it is an explanation. The secret is to tell [the patient] things first*”.

## Discussion

This study explored the phenomenon of “difficult” patients in dental care from the caregiver’s point of view. The meaning of “difficult” dental patients was highlighted in the core category *balancing subjective difficulties*, followed by seven descriptive interrelated lower-level categories grounded in the data. The theoretical framework indicated patient characteristics and their influencing effects on dental staff, as well as the problem-solving strategies staff employed to manage these patients and their treatments (Figs. 1 and 2).

Compared to previous research in other health care arenas, where relationships between specific diagnoses and difficult behaviors have been explored [4], this study proposed a dynamic and complex process through which a patient may be perceived as “difficult”. This process not only depends on the patients’ characteristics and affecting abilities but also on the dental staff’s perceptions of the patients’ expressions. Thus, the dental caregivers seem to not only to respond to “difficulties”, but they actively modify their own experiences before, during, and after interactions with “difficult” patients. This study indicated that the staff’s experiences of patient characteristics and how patients affected them varied depending on the care providers’ own internal resources and abilities to receive, manage, process, and host the patients’ expressions, as well as on their previous experiences of encounters with and treatment of “difficult” patients, and the support they received. Together, this modified and balanced the perceptions that formed the “difficult” patient phenomenon: What is experienced as difficult by one caregiver does not have to be difficult for another.

The specific abilities and skills used by dental staff to ease the communication with, and treatment of, patients have been described in previous research and include adaptive interpersonal skills among dentists, such as active listening, empathic understanding, verbal and non-verbal communication, conveying control and confidence, and being a fellow human being [30, 31]. In our study, the process of getting to know a patient to understand their needs and expectations was facilitated by the participants’ use of their own personal recourses, balanced with their professionalism. This could be an energy-draining and time-consuming process where the caregivers have to balance themselves between nearness and distance, and compassion and empathy, with personal and professional skills. This approach lies near the definition of person-centered care, which has recently been discussed as one of the skills that will shape the future of dentistry [32, 33]. A person-centered care model focuses on the elements of care, support, and treatment that matter to the patient and is applied in collaboration with them [34].

We interpreted the core category *balancing subjective difficulties*, by activating the use of resources and creating adaptive treatment relations, as suggestions for problem-solving regarding the main concern (Fig. 2). In the present study, the professional treatment of “difficult” patients required an empathic attitude and the ability to show and feel nearness to the patient while also maintaining a certain distance from, and perspective on, the patient and the treatment situation so as to process their expressions and set professional and personal boundaries. Compassion toward “difficult” patients might include learning, which could give the dental staff a more secure role interpretation, knowledge about social-skills, and easier access to individual well-functioning handling strategies. One example of this learning through self-reflection was the “thinking through, learning, and letting go” -process described by our participants, which pointed the dental staff toward a self‐conscious narration of caregiving.

Additionally, the result may be understood through the dental staff’s experiences of time pressure. However, the findings revealed that lack of time with “difficult” patients was not the only explanatory factor for the characteristics and predictors of the experienced “difficulties”. The resulting framework indicates a range of factors as to why a patient may be perceived as “difficult”. Challenging encounters seem to stress and frustrate professional caregivers but also, on the contrary, cultivate reflection, leading to a beneficial deepened knowledge about one’s strengths, and ongoing professional development and learning. This may be understood as important for the dental staff’s professional role-taking in the long-term. But to generate these potential positive outcomes stress the necessity for sufficient time and focus to bring the patient and/or oneself at the center of attention.

This study’s limitations relate to the nature of qualitative research, with its theory-generating approach and collection of so-called “soft data”, such as experiences, that are not directly measurable or projectable. The conclusions were built upon the participants’ experiences and the researchers’ interpretation of these experiences rather than on statistical calculations. The study’s data were collected from the same public dental health institution (public dental service in the Västra Götaland region of Sweden). Another limitation relates to our aim: to elucidate the phenomenon from *the dental staff’s* point of view. A different aim, from the patient perspective, might have provided opportunities for other discoveries. The term “difficult” patient may be perceived as a bit challenging, but it has relevance as a label in day-to-day discourse in dental offices during clinical practice. Other definitions of such patients may be of importance, such as “challenging encounters” and “problematic interactions”. But these labels risk falling short in our study aimed to illuminate the experiences related to the clinical cultural context of dentistry. Another important challenge was to ensure the participants’ tone of voice during translation from Swedish to English. All these issues relate to the nature of a qualitative method, which requires a considerable amount of deliberate performance from the research team.

The strength of the study lies in the fact that the result is grounded in the empirical data of the experiences of the interviewed staff who perform dental care on “difficult” patients (the theory has fit) and that it explains the phenomenon under study (the theory works). The constant comparative analysis process contributed to the quality of the data through strategically sampling participants and receiving saturation in data. Fair use of citations from the participants, rich descriptions of the results and their context, and the continuous reflective standpoint taken by the authors in not letting pre-understanding influence the collection and analyze during the research process enable the reader to conclude the transferability and trustworthiness of the study.

Our definition of “difficult” patients adds a focus on resilience by *balancing subjective difficulties* as an important part of the “difficult” patient experience. Thus, the dental caregivers seem not only to respond to “difficulties”, but they are an active modifier of their experiences before, during, and after interactions with “difficult” patients. More research is needed to further explore how an adaptive treatment relationship may be built and maintained. Future research could target potential preventive behaviors and factors for resilience (i.e., compassion competence, emotional self-regulation, or communication skills) among dental health professionals so as to make them better equipped to both prevent and cope with challenging clinical interactions. This may, in the long run, assist our profession to utilize person-centered care.

## Conclusion

The dental staff’s meaning of the phenomenon of “difficult” dental patients point to specific characteristics, effects, and handling strategies. The core category of the main concern emerged as “*balancing subjective difficulties*”. “Difficult” patient characteristics and affects are dynamic concepts related to the individual caregivers’ handling capacity in an ongoing context. This indicates a need for further in-depth studies regarding dental interaction and studies generalizing the outlook on “difficult” dental patients.
